# What Clinical Information Is Valuable to Doctors Using Mobile Electronic Medical Records and When?

**DOI:** 10.2196/jmir.8128

**Published:** 2017-10-18

**Authors:** Junetae Kim, Yura Lee, Sanghee Lim, Jeong Hoon Kim, Byungtae Lee, Jae-Ho Lee

**Affiliations:** ^1^ School of Management Engineering Korea Advanced Institute of Science and Technology (KAIST) Seoul Republic Of Korea; ^2^ Department of Biomedical Informatics Asan Medical Center University of Ulsan College of Medicine Seoul Republic Of Korea; ^3^ Carey Business School The Johns Hopkins University Baltimore, MD United States; ^4^ Medical Information Office Asan Medical Center Seoul Republic Of Korea; ^5^ Department of Emergency Medicine Asan Medical Center University of Ulsan College of Medicine Seoul Republic Of Korea

**Keywords:** mobile health, electronic medical records, clinical information, rounding, timeliness, accessibility, smartphone

## Abstract

**Background:**

There has been a lack of understanding on what types of specific clinical information are most valuable for doctors to access through mobile-based electronic medical records (m-EMRs) and when they access such information. Furthermore, it has not been clearly discussed why the value of such information is high.

**Objective:**

The goal of this study was to investigate the types of clinical information that are most valuable to doctors to access through an m-EMR and when such information is accessed.

**Methods:**

Since 2010, an m-EMR has been used in a tertiary hospital in Seoul, South Korea. The usage logs of the m-EMR by doctors were gathered from March to December 2015. Descriptive analyses were conducted to explore the overall usage patterns of the m-EMR. To assess the value of the clinical information provided, the usage patterns of both the m-EMR and a hospital information system (HIS) were compared on an hourly basis. The peak usage times of the m-EMR were defined as continuous intervals having normalized usage values that are greater than 0.5. The usage logs were processed as an indicator representing specific clinical information using factor analysis. Random intercept logistic regression was used to explore the type of clinical information that is frequently accessed during the peak usage times.

**Results:**

A total of 524,929 usage logs from 653 doctors (229 professors, 161 fellows, and 263 residents; mean age: 37.55 years; males: 415 [63.6%]) were analyzed. The highest average number of m-EMR usage logs (897) was by medical residents, whereas the lowest (292) was by surgical residents. The usage amount for three menus, namely inpatient list (47,096), lab results (38,508), and investigation list (25,336), accounted for 60.1% of the peak time usage. The HIS was used most frequently during regular hours (9:00 AM to 5:00 PM). The peak usage time of the m-EMR was early in the morning (6:00 AM to 10:00 AM), and the use of the m-EMR from early evening (5:00 PM) to midnight was higher than during regular business hours. Four factors representing the types of clinical information were extracted through factor analysis. Factors related to patient investigation status and patient conditions were associated with the peak usage times of the m-EMR (*P*<.01).

**Conclusions:**

Access to information regarding patient investigation status and patient conditions is crucial for decision making during morning activities, including ward rounds. The m-EMRs allow doctors to maintain the continuity of their clinical information regardless of the time and location constraints. Thus, m-EMRs will best evolve in a manner that enhances the accessibility of clinical information helpful to the decision-making process under such constraints.

## Introduction

Clinical work that takes place in various locations (ie, wards or clinics) and involves various treatment tasks (ie, diagnosis or operation) requires doctors to move a lot [1,2]. Mobility is a particularly important feature of clinical practice in large medical institutions with complex treatment procedures [1]. Therefore, mobile-based electronic medical records (m-EMRs) have been expected to help doctors efficiently access patient data [1,2], and many tertiary hospitals have increasingly moved toward the use of m-EMRs in recent years [3-5]. However, because the overall rates of m-EMR utilization and adoption have been low [3,6], several studies have been conducted to improve the usability of m-EMRs in hospitals [3-8].

One research stream examined the behavioral patterns related to the adoption and use of m-EMRs, including personality traits and social norms [3,6]. Another research stream studied the design of m-EMR systems and their integration with existing hospital systems [4,7,8], whereas another focused on demonstrating the utility of m-EMRs with regard to information flow efficiency [9-11]. Such studies have certain implications in that they examined the theoretical and technical factors associated with the adoption and utilization of m-EMRs and demonstrated that m-EMRs increase the work efficiency. However, to the best of the authors’ knowledge, none of these previous studies have evaluated the value of each type of clinical information accessed through m-EMRs based on actual usage log data. Because an m-EMR is a method of information delivery, an evaluation is crucial for designing m-EMRs in a manner that allows doctors to access valuable information in a convenient manner.

Typically, clinical work is carried out through a daily process, which is organized based on hospital conditions [12,13]. Because each process unit requires different tasks from the doctors, the demand for information access may vary according to the daily process unit [1,12]. In addition, the need for specific clinical information related to the treatment context may vary within the daily process. So, it is important to assess the value of clinical information accessed through m-EMRs from the perspective of the daily treatment process. Despite the importance of m-EMRs, there have been no attempts at exploring when the value of m-EMR usage increases during the day and what clinical information is associated with its increased value.

These attempts may provide fundamental solutions for increasing the use of m-EMRs in large hospitals by identifying the most valuable clinical information accessed through such records. Additionally, these discussions may provide knowledge in research areas investigating the value of m-EMR usage in terms of information flow efficiency. Therefore, as a first attempt to shed light on the issues mentioned above, this study aimed to explore an empirical resolution on what type of clinical information is most valuable for doctors to access through an m-EMR based on their actual usage logs and when such information is accessed. In addition, this study aimed to discuss the importance of such information.

## Methods

### Introduction to m-EMR App

A tertiary hospital in Seoul, South Korea, with more than 2700 beds and approximately 912,300 admissions each year developed an m-EMR app in 2010. The main purpose of this m-EMR app is to allow medical personnel to read patient information without issuing treatment orders [14]. The second version of the upgraded m-EMR app, based on user feedback in 2012, was used in this study. An add-on security system temporarily displays clinical information without storing the information on a smartphone device.

**Figure 1:**
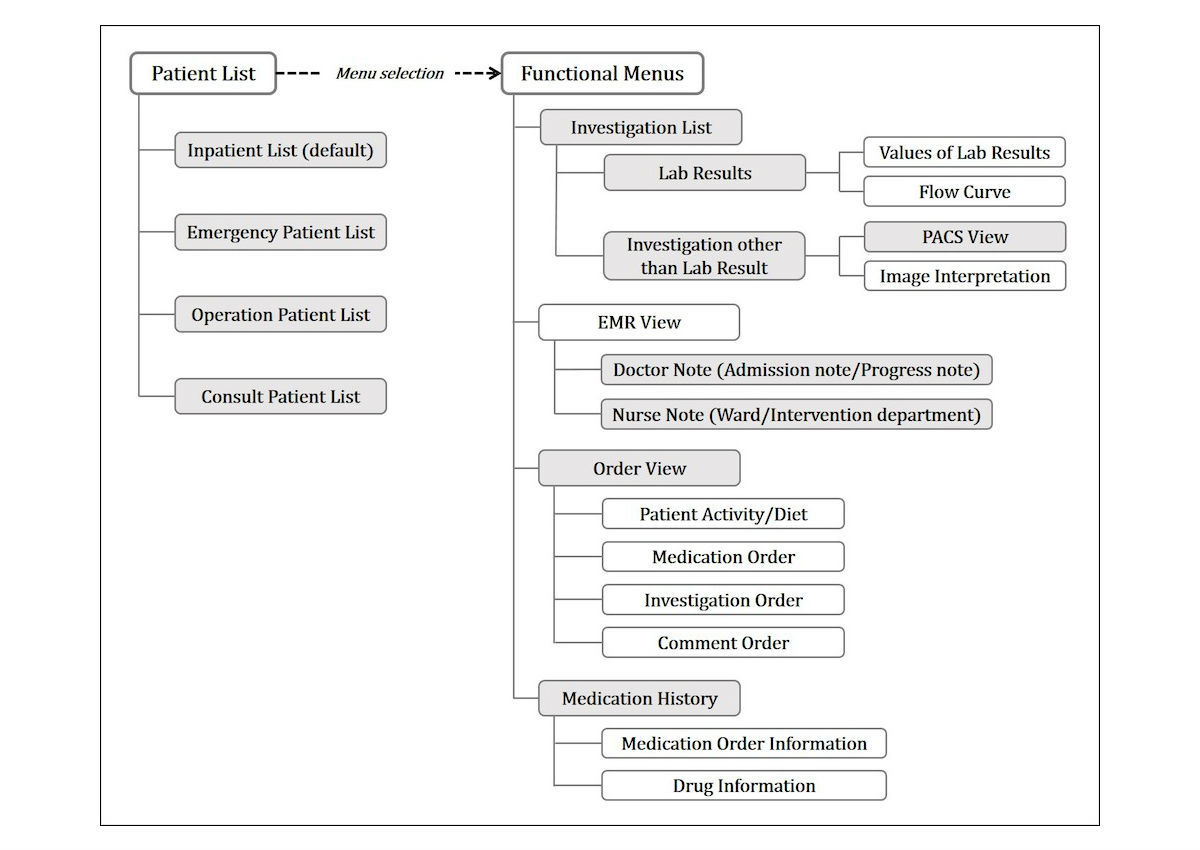
Structure of information accessed through the hospital’s mobile-based electronic medical records app. Usage logs from 12 menus (gray-shaded menus) providing 22 types of information were used in this study. PACS means picture archiving and communication system.

The app comprises four default menus and several submenus. The default menus provide patient lists, and doctors can choose one of the following menus: inpatient list, operation patient list, consult patient list, and emergency patient list. The submenus allow doctors to access patient details such as laboratory test results, medical records, and medication orders. The structure of information accessed through the m-EMR app is shown in Figure 1 (see the details on the m-EMR app in Multimedia Appendix 1).

### Empirical Analysis Design

This research was approved by the institutional review board (IRB No. 2016-0287). To determine what type of clinical information is most valuable for doctors to access through an m-EMR and when such information is accessed, a two-step empirical analysis was conducted. First, the usage patterns of both the m-EMR and the hospital information system (HIS) on an hourly basis were explored. Comparing the usage patterns for both types of systems can provide an explanation on when access to clinical information through m-EMRs is valuable. Furthermore, it can provide a basis to explain why certain clinical information read through an m-EMR is more valuable than when read using the HIS.

Second, the types of clinical information accessed most frequently during m-EMR peak usage times were investigated. The usage concentration of a particular type of information within a specific time interval indicated that its value was high at that time [15]. Therefore, associating the peak intervals of usage with specific clinical information can explain what types of clinical information are most valuable to access through m-EMRs.

When evaluating clinical information, it might be inappropriate to analyze the m-EMR usage logs at a very raw level (ie, usage count of each menu). Although some menus are used frequently, they may serve as intermediary channels to reach submenus that access detailed information. Thus, it is important to mine the raw usage logs so that usage patterns become representational clinical information. Data preprocessing and factor analysis were applied to extract representational clinical information. Finally, a random intercept logistic regression was employed to determine the association between usage peak intervals and representational clinical information.

**Figure 2:**
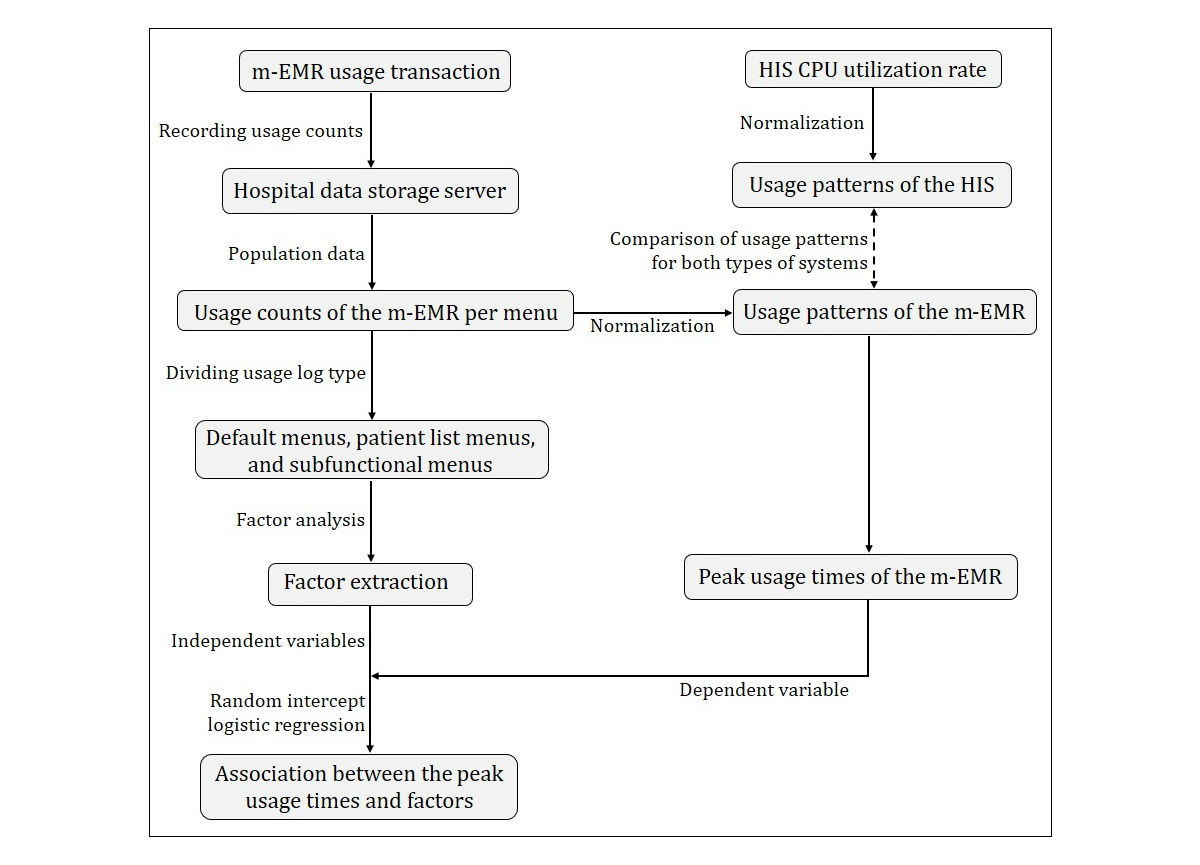
Flowchart of data preprocessing and analysis; m-EMR: mobile-based electronic medical records, HIS: hospital information system, CPU: central processing unit.

For the study data, usage counts (population data) of the m-EMR and the utilization rate of the HIS central processing unit (CPU) were used. The CPU usage rate represents the amount of time that the CPU processes tasks in a specific time interval [16]. The HIS CPU processes tasks when requests are made to read patient information from a local personal computer (PC). Thus, the HIS CPU usage rate indicates how often doctors read clinical information through a desktop computer during specific time intervals. Figure 2 provides a flowchart illustrating the data preprocessing and analysis.

### Data Preprocessing for Mining m-EMR Usage Patterns

The structure of the m-EMR was designed to display some lower-level information (ie, lab result values) simultaneously using upper-level information (ie, lab results) (Figure 1). The hospital data storage server records the usage transactions for each of the m-EMR menus when the app menu is used.

Owing to their default status, the four patient list menus are likely to be used regardless of intent. Thus, the usage amount of these menus should be treated differently from that of the other submenus, even though these menus provide the function of a patient list check. To address this issue, logs used primarily to check patient lists (the four patient list menus) were separated from logs used to access detailed patient information. Specifically, if the log remained in the four default menus (ie, there were no usage traces after these default menus had been used) during one usage session, it was considered that the doctor simply identified the patient lists during that session. However, if there were traces indicating that the submenus were used after the four default menus had been used, it was considered that the doctor accessed detailed information. Thus, the four patient list menus could each have had two purposes (four menus × two purposes). Therefore, 16 variables representing the usage logs of the menus were included in this study (four patient list menus assumed to be default menus used to access submenus, designated by the subscript “default”; four patient list menus assumed to be used to check patient lists; and eight submenus). R version 3.3.2 (The R Project for Statistical Computing) was used for data preprocessing.

### Descriptive Analysis of Usage Patterns of m-EMR

First, the general usage statistics of the medical and surgical departments were reviewed to determine whether m-EMR use differed according to the user characteristics and tasks. Second, the usages of the m-EMR and the HIS CPU over time were compared. The units of the two usage logs are different because the m-EMR usage level is based on the usage counts, whereas the HIS CPU usage level is based on the CPU utilization rate. Thus, the normalized values of the HIS and m-EMR usage over time were compared. Third, the peak usage intervals of the m-EMR were defined. The usage counts (number of times the m-EMR was accessed) per hour were normalized, and a continuous interval with normalized values that are greater than 0.5 (ie, the median of the normalized values) was defined as a peak interval. Details of the usage per menu during the peak usage interval were then examined at the raw-data level.

### Factor Analysis: Identification of Representational Clinical Information

In a hierarchical app design, higher-level menus serve as links to the submenus while providing particular information [3,8]. Therefore, usage logs for some upper- and middle-level menus might not adequately represent a doctor accessing particular information from the m-EMR menu. Thus, the usage logs were partitioned into usage session units, and indicators representing how closely a usage session is associated with specific clinical information were parameterized. A usage session for a smartphone app represents the interval between the time an app is launched and the time it is closed [17-19]. To identify a usage session, usage logs are separated into 30-min intervals set in the hospital system to force an automatic m-EMR app log-off.

To generate indicators of how relevant a usage session is to specific clinical information (ie, representational clinical information), a factor analysis was applied [20-22]. There were 16 variables applied to this analysis to indicate the usage level of the menus during a usage session. A principal component analysis was used to extract the factors [20,21]. The promax rotation method was used to rotate the factors because this method is recommended when factors might have certain correlations [22]. The factors were extracted until the communality of all variables was greater than 0.4, and variables with the lowest communality values were excluded [23]. In addition, only factors with eigenvalues greater than 1 were extracted [24]. To assess the validity of the factor analysis, a Keiser–Meyer–Olkin test and a Bartlett test were applied [25-27]. SPSS version 23 (IBM Corp) was used for the factor analysis. A detailed description of this factor analysis has been provided in previous studies [20-27].

### Analysis of Frequently Accessed Clinical Information During Peak Usage Intervals

**Figure 3:**
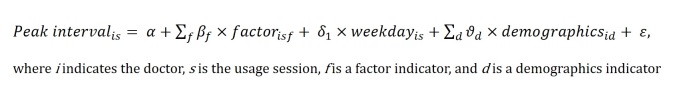
Equation for random intercept logistic regression.

To analyze what type of clinical information is accessed frequently during peak m-EMR usage intervals, a random intercept logistic regression was applied. The random intercept model is often used to address individual heterogeneity when data are observed repeatedly [28]. The random intercept logistic model in this study is designed as shown in Figure 3.

The dependent variable (1=peak usage time, 0=outside the peak usage time) indicates whether a usage session belonged to the usage peak interval of the m-EMR. For the independent variables, the scores from the results of the factor analysis were used. In addition, the model controlled whether the m-EMR was used on a weekday or holiday, and for the demographics, that is, age, gender, and six positions (residents, fellows, and professors from medical departments and residents, fellows, and professors from surgical departments). The model was implemented using STATA version 14 (StataCorp LLC).

## Results

### Descriptive Analysis

A total of 524,929 usage logs for 12 menus, which provide 22 types of information, were stored during the study period (March to December 2015). The overall user characteristics and usage statistics are listed in Multimedia Appendix 2. When simultaneously considering the medical and surgical departments, the mean usage counts for professors, fellows, and residents were 732, 754, and 897, respectively. For the medical departments, the mean usage counts for doctor positions were 789, 865, and 1216, respectively, and 656, 594, and 292 for the surgical departments, respectively. Therefore, the m-EMR was used the most by medical residents, whereas the individual average usage of the m-EMR by the surgical residents was the least.

**Figure 4:**
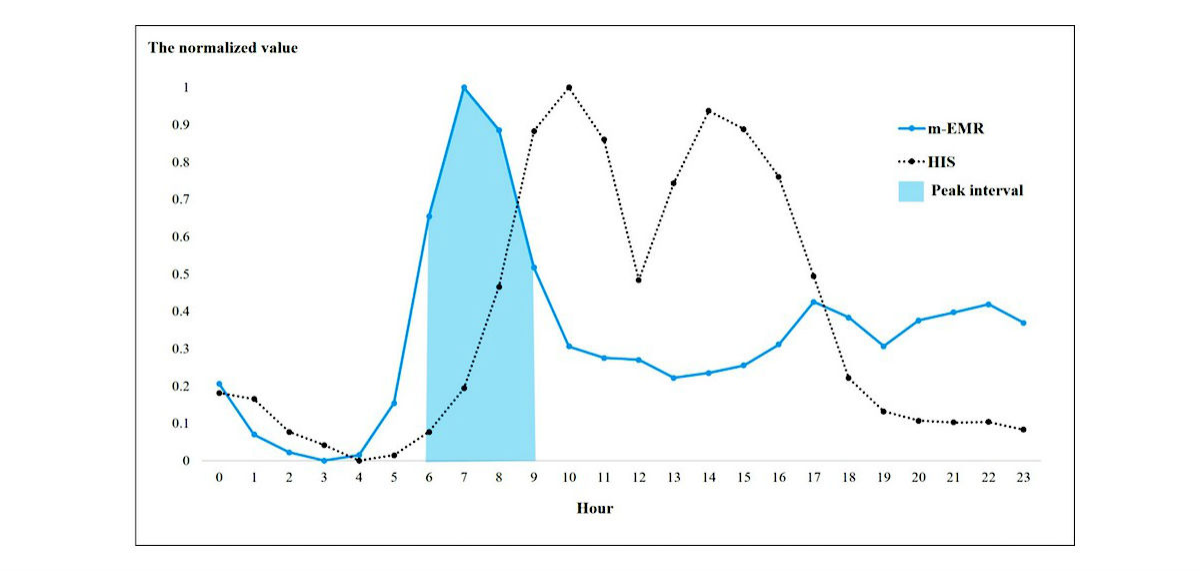
Difference in peak times between the m-EMR (mobile-based electronic medical records) and HIS (hospital information system). The graph of the m-EMR shows the normalized values over time, based on the m-EMR usage log. The graph of the HIS indicates the normalized values over time, based on the HIS CPU utilization rate. Each unit on the x-axis represents the hour (ie, 9 indicates the hour between 9:00 AM and 10:00 AM.).

The HIS CPU usage rate for one week of November 2016 was used in this study. The usage patterns of both the HIS and the m-EMR based on the time of day were significantly different (Figure 4). The use of the HIS was highly concentrated during regular business hours. The HIS was used most frequently at two different periods: the first from approximately 9:00 am to 12:00 pm and the second from approximately 1:00 pm to 5:00 pm. In contrast to the usage patterns for the HIS, the m-EMR was heavily used during the early morning hours (6:00 am to 10:00 am). Moreover, the usage rate of the m-EMR from early evening (5:00 pm) to midnight (0:00 am) was higher than that during regular business hours.

The peak usage interval for the m-EMR was defined as 6:00 am to 10:00 am. Table 1 lists the details of the per-menu usage statistics during the usage peak interval in descending order. The most commonly used menus include the inpatient list (47,096), lab results (38,508), and investigation list (25,336). The usage amounts of these three menus accounted for approximately 60.1% of the peak time usage.

**Table 1:** Usage statistics of the m-EMR menus at peak usage intervals.

Usage count	Time				Total
	6-7 am (n=357)	7-8 am (n=460)	8-9 am (n=474)	9-10 am (n=429)	(6-10 am)
Inpatient list	10,059	15,207	13,681	8149	47,096
Lab results	5810	10,051	12,818	9829	38,508
Investigation list	3668	7156	8636	5876	25,336
Doctor note	6083	5587	4193	2088	17,951
Nurse note	7654	5655	2581	1196	17,086
Investigation other than lab results	2169	5285	5134	2339	14927
PACS (picture archiving and communication system) view	1639	2661	2586	1324	8210
Order view	1073	2352	1430	724	5579
Consult patient list	1379	1718	1168	506	4771
Emergency patient list	816	1042	937	538	3333
Operation patient list	219	856	323	257	1655
Medication history	15	54	54	28	151

### Results of Factor Analysis: Identification of Representational Clinical Information

A total of five factors with 13 variables were extracted under the conditions that the eigenvalues were greater than 1 and that the communality value for all variables was greater than 0.4 (Table 2) [23,24]. The results of the two tests, Keiser–Meyer–Olkin test (0.663) and Bartlett test (*P*<.01), indicated the validity of the factor analysis [25-27].

Factor 1 (F1): investigation status. This indicates a session in which a doctor accesses the investigation status and is defined based on a positive association with the variables of investigations (Table 2).

Factor 2 (F2): emergency patient information. This indicates a session in which a doctor accesses emergency patient information and is defined based on a positive association with the Emergency patient list_default_ and Doctor note variables.

Factor 3 (F3): patient conditions. This indicates a session in which a doctor accesses previous patient conditions and is defined based on a positive association with the Nurse note and Order view variables.

Factor 4 (F4): identification of patients in the emergency room (ER) or ward. This indicates a session in which a doctor identifies a patient in the ER or ward and is defined based on a positive association with the Emergency patient list and Inpatient list variables.

Factor 5 (F5): miscellaneous. This indicates a session in which the information access does not show a clear pattern. These sessions are associated with default menus and are indications that the doctor is accessing patient details through the submenus. However, because no usage patterns of the submenus can be determined, sessions associated with this factor are considered as miscellaneous.

**Figure 5:**
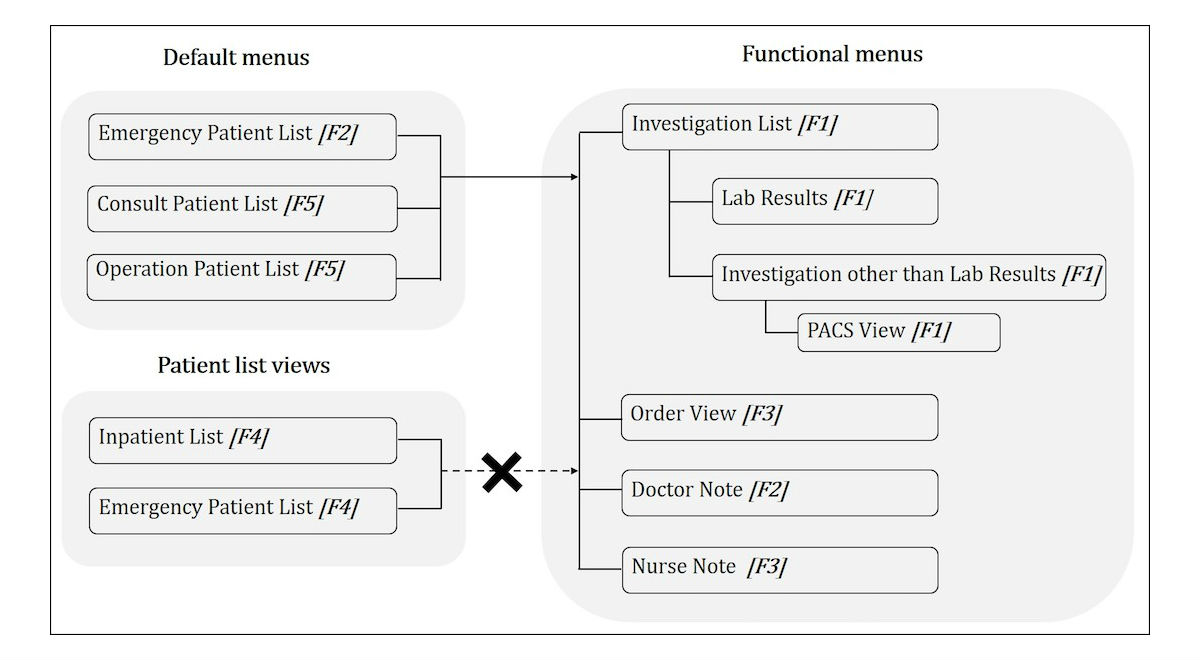
Diagram of associations between factors (only factors with loading values greater than 0.4 are listed); PACS: picture archiving and communication system.

None of the factors have a strong relationship (ie, factor loading with an absolute value greater than 0.4) with the Inpatient list_default_ variable. This indicates a lack of correlation between Inpatient list_default_ and other menu uses during a single usage session. Figure 5 shows the association between these five factors, and Table 3 provides brief descriptions of them.

**Table 2:** Results of factor analysis.

Variables	Factor					Communality^e^
	F1	F2	F3	F4	F5	
Investigation other than lab results	*.809*^a^	.050	−.022	.204	.096	.603
PACS (picture archiving and communication system) view	*.793*	−.017	−.120	.126	.060	.549
Investigation list	*.750*	.016	.078	−.119	−.087	.693
Lab results	*.465*	−.173	.120	−.281	−.199	.460
Emergency patient list_default_^b^	−.003	*.944*	−.220	.021	.011	.808
Doctor note	−.041	*.730*	.376	.044	−.079	.800
Nurse note	−.140	.075	*.815*	.044	−.030	.649
Order view	.109	−.147	*.753*	.100	.107	.544
Emergency patient list	.200	.064	.099	*.793*	−.027	.529
Inpatient list	−.023	−.030	.067	*.742*	−.053	.552
Inpatient list_default_	.227	.347	.066	−.375	.103	.516
Operation patient list_default_	.070	.053	−.136	−.004	*.714*	.541
Consult patient list_default_	−.049	−.099	.264	−.100	*.700*	.544
Result of adequacy tests for factor analysis	Bartlett test^c^: *P*<.01					
	Keiser–Meyer–Olkin test^d^: 0.663					

^a^Factor loadings with absolute values greater than 0.4 are in italics.

^b^The “default” subscript indicates a menu likely used as the default screen.

^c^Bartlett test evaluates the presence of a common component.

^d^The Keiser–Meyer–Olkin test evaluates the appropriateness of the size of observations and number of variables used in the factor analysis.

^e^Communality indicates how much the extracted factors account for each variable.

### Analysis of Frequently Accessed Clinical Information During Peak Usage Interval

The results of a random intercept logistic regression indicate that F1 (investigation status) and F3 (patient conditions) are positively associated with peak usage intervals (*P*<.01) (Table 3). By contrast, F2 (emergency patient information), F4 (identification of patients in the ER or ward), and F5 (miscellaneous) are positively associated with periods outside the peak usage intervals (*P*<.01).

The control variable, Weekday, is statistically significant (*P*<.01), indicating that usage sessions on weekdays are positively associated with the peak intervals. In addition, the usage sessions of doctors other than surgical residents are more positively associated with the usage peak than those of surgical residents (*P*<.05). Age and gender are not statistically associated with the usage sessions at the peak usage intervals (*P*>.05).

**Table:** 

Variable				Coefficient		Standard error		*P* value
**Main variables**								
	F1 (investigation status)		.038		0.011		.001	
	F2 (emergency patient information)		−.226		0.017		<.001	
	F3 (patient conditions)		.210		0.013		<.001	
	F4 (identification of patients in the emergency room or ward)		−.109		0.013		<.001	
	F5 (miscellaneous)		−.126		0.014		<.001	
**Control variables**								
	Weekday		.566		0.023		<.001	
	**Position^a^**							
		Fellows (general medical departments)	.667		0.126		<.001	
		Fellows (surgical departments)	.417		0.146		.01	
		Professors (general medical departments)	.503		0.153		<.001	
		Professors (surgical departments)	.440		0.166		.01	
		Residents (general medical departments)	.302		0.111		.01	
	Age			−.008		0.006		.22
	Gender			.0240		0.073		.75
	Cons			−1.445		0.216		<.001

^a^The rank of residents from surgical departments was used as the baseline position to control the doctor position characteristics. The dependent variable indicates whether the usage session belongs to the peak interval or lies outside the usage peak interval (1=peak usage, 0=outside the peak usage). The number of observations is 56,756 (usage sessions), and the number of doctors is 653.

## Discussion

This study aimed to explore what types of clinical information accessed through an m-EMR are most valuable for doctors and when they access such information and to discuss how valuable such clinical information actually is. In large hospitals with complex treatment processes, patient care necessarily entails significant doctor movement. In such an environment, continuous awareness of the patient information through a desktop PC may not be efficient for doctors. Thus, several previous studies have demonstrated the utility of using mobile devices in relation to information flow efficiency during the treatment process [9-11]. However, there has been no empirical assessment on the value of clinical information from the viewpoint of routine treatment, which provides a fundamental explanation as to what type of valuable clinical information is accessed through m-EMRs and when. Therefore, this study is distinct from previous studies in that, to the best of the authors’ knowledge, it is the first attempt to evaluate clinical information accessed through an m-EMR from large real-usage data. Ultimately, this study may contribute to promoting the adoption and usability of m-EMRs in large hospitals by providing some important insights.

### Location Independence in Accessing Information Through m-EMRs

The analysis conducted in this study demonstrates the unique value of an m-EMR system, which is distinct from a PC-based system in terms of information transaction. Interestingly, the m-EMR appears to be used frequently at times when the HIS is rarely used. Specifically, the HIS is heavily used during regular business hours (9:00 am to 6:00 pm), whereas the use of the m-EMR peaks early in the morning (6:00 am to 10:00 am). The m-EMR usage peak corresponds to morning rounds or the time just before routine work begins [29,30]. During this time, access to patient information is necessary, but information accessed through a desktop PC can be limited because the doctors should move around a great deal (ie, commuting and conducting ward rounds) [11,31]. Earlier studies have shown that the use of mobile devices during ward rounds is effective with regard to information acquisition because mobile devices provide doctors with location-independent access to information [11,32]. Consistent with this evidence, the results of this study may indicate that doctors use m-EMRs intensively to identify patient information during their ward rounds. Moreover, this result suggests that doctors use m-EMRs to read patient information even before and during their morning rounds.

Furthermore, the use of the m-EMR is higher from early evening (5:00 pm) to midnight than during regular business hours. The high usage rate of the m-EMR during this time may indicate that doctors outside the hospital access patient information through the system. Owing to the continuity of patient care, doctors should check their patient information after work or share their opinions with colleagues who are on the night shift [33]. However, it is very troublesome for doctors to return to the hospital to check their patient information. In this regard, m-EMR can be a valuable tool that allows them to access such information regardless of location and time constraints. Therefore, the results of this analysis further strengthen the evidence that m-EMRs are valuable to doctors in terms of location-independence when accessing clinical information.

### High Demand for Data Science Skills to Explore m-EMR Usage Patterns

The results of this study indicate that an analysis of raw-level usage logs might lead to distorted results when exploring m-EMR usage patterns. Owing to the nature of the m-EMR structure, some menus can often be used regardless of intent. For instance, the inpatient list as one of the default menus is most frequently used during the peak usage interval at the raw-data level. There are two purposes for using this menu. First, the menu can be used as a simple patient checklist to review a list of patients under the doctor's responsibility or a list of newly admitted patients. Second, the menu can be unintentionally used owing to the default state of the menu. Considering the entire analysis, most doctors in this study might have set the inpatient list as their default screen. Specifically, the results of a descriptive analysis show that the use of the inpatient list was overwhelming, in contrast to the low use of other candidate default menus (ie, consult, emergency, and operation patient lists). Given that doctors have to use the default menu before using other submenus of the m-EMR app, its high utilization may indicate that the inpatient list menu is used most frequently as the default menu. Moreover, the results of a factor analysis indicate that there is no clear usage pattern after the Inpatient list_default_ has been used. These results suggest that the inpatient list is used frequently as the default screen regardless of the doctor's intention. In addition, the investigation list is a gate menu located at the middle level for grouping the investigation results of patients rather than providing specific clinical information. Although the usage of these menus is high (ie, the first and third most frequently used menus), their usage amount may not be crucial in assessing the value of specific clinical information accessed through an m-EMR. These facts emphasize the importance of data science skills when examining the usage features of m-EMRs. Several advanced data mining techniques can be useful to investigate the usage characteristics of m-EMRs in more detail. For instance, process and sequential mining techniques may provide a better explanation on how doctors use m-EMRs by identifying and visualizing the sequence of usage patterns [34,35].

### Information on Patient Investigation Status and Conditions That Help With Decision Making During Ward Rounds

This study found four patterns of representational clinical information access (ie, investigation status, patient conditions, emergency patient information, and identification of patients in the ER or ward) when using an m-EMR. These differentiated usage patterns might indicate that specific information was accessed in an m-EMR usage session according to the treatment context. In other words, it might indicate that the m-EMR was used for unique purposes during each usage session. According to a regression analysis, the investigation status and patient conditions are positively associated with the times of peak usage, which correspond to the morning rounds or the time just before the rounds begin. Previous studies showed that important decisions in a treatment environment are made during the ward rounds [31,36,37]. To make a correct decision, it is important to have discussions based on the specific clinical information according to the treatment context. Information on the investigation results and patient progress records is known to be crucial to the decision-making process [31,36,37]. The information is associated with the investigation status (investigation other than lab results, PACS [picture archiving and communication system] view, investigation list, and lab results) and patient conditions (nurse note and order view) based on a factor analysis conducted in this study. Information access through a desktop PC is likely limited during the early hours at approximately the time of morning rounds. Thus, using a desktop PC to keep track of an investigation status and the conditions of the patients may not be convenient for doctors. Under such circumstances, m-EMRs can help doctors to communicate with their colleagues for information sharing or discussions by providing immediate access to the investigation status and patient conditions. Hence, the results of this study suggest that access to the investigation status and patient conditions through m-EMRs is highly valuable to doctors in terms of decision making during the time ward rounds are conducted.

### Different Needs for Accessing Information Through m-EMR Depending on Department

The results of this study suggest that information obtained by a doctor through an m-EMR varies depending on the doctor’s department or task. A descriptive analysis shows that the overall usage of the m-EMR by doctors in general medical departments is higher than that of doctors in surgical departments. These results can be explained in terms of the intrinsic differences between the medical and surgical departments. Although both groups of doctors have the common goal of treating their patients, their tasks and working environments are different [38]. Specifically, because the doctors in surgical departments often have important tasks in an operating room [38], they may have already experientially shared important information when they were there. Additionally, they often obtain information through direct patient contact such as physical investigations or wound dressing. By contrast, doctors in medical departments often work by examining the patient's condition or interpreting the patient's diagnosis based on various types of information [31]. These differences between the two groups may constitute different needs for information and different preferences for the way the information is acquired. Therefore, doctors in surgical departments may use an m-EMR only to acquire key patient information. On the other hand, doctors in medical departments have a high demand for reviewing and sharing patient information with other colleagues. In this regard, information access through m-EMRs can be more valuable to doctors in medical departments than doctors in surgical departments.

### Limitations

This research has several limitations. First, the research was conducted using log data from an m-EMR app used in only a single hospital. It is likely that each hospital has a unique m-EMR system and different schedules for its ward rounds. Therefore, other research environments might yield different results from those of this study. However, the value of an m-EMR in terms of information access is expected to also be demonstrable in other research environments. Second, it is acknowledged that more data are required to enable much better research. The data collection period for the m-EMR usage in this study differed from that for the HIS CPU usage rate. However, considering that the medical staff do not significantly change the way they use the HIS during their work processes, an analysis using log data from the m-EMR app and the HIS during the same period is expected to yield results similar to those of this study. In addition, information on personal and organizational tendencies regarding the use of m-EMRs was not included in this study. Previous studies have shown that personal and organizational characteristics have significant impacts on information technology usage in hospitals [3,39-42]. Therefore, using this information for analysis is expected to improve the robustness of this research stream. Third, this study focused only on information read through an m-EMR and did not consider information entries. It would be valuable to examine whether the investigation status or patient conditions are not frequently recorded through m-EMRs during morning ward rounds.

### Conclusions

The most prominent feature of an m-EMR is location-independence in terms of information accessibility. Thus, m-EMRs can be best designed to facilitate access to information when doctors are under time and location constraints. Particularly during the early morning when access to clinical information through a desktop PC is highly limited, doctors can read information regarding a patient’s status using an m-EMR. In this regard, m-EMRs will best evolve in such a way that patient information essential for decision making during ward rounds is easily accessed and effectively presented.

Further research is required to gain a deeper understanding of m-EMR usage. The requirements for information acquisition through an m-EMR may vary according to the characteristics of different medical tasks. In addition, clinical information can be presented in various ways, depending on the design of particular m-EMRs. Thus, there may be research opportunities in exploring representational clinical information in other medical environments or using other m-EMR designs. Additionally, further research may aim to investigate the association between specific doctor groups and preferences for the types of information accessed through an m-EMR.

## Appendix

Multimedia Appendix 1 Service Structure and Contents of the mobile electronic medical record.

Multimedia Appendix 2 Overall usage statistics of the m-EMR based on doctor position.

## Abbreviations

CPU: central processing unit
ER: emergency room
HIS: hospital information system
m-EMR: mobile electronic medical records
PACS: picture archiving and communication system
PC: personal computer
