# Supplementary figures and images for: What Clinical Information Is Valuable to Doctors Using Mobile Electronic Medical Records and When?
Source: J Med Internet Res. 2017 Oct 18;19(10):e340. doi: 10.2196/jmir.8128 (PMC5666226; doi:10.2196/jmir.8128)

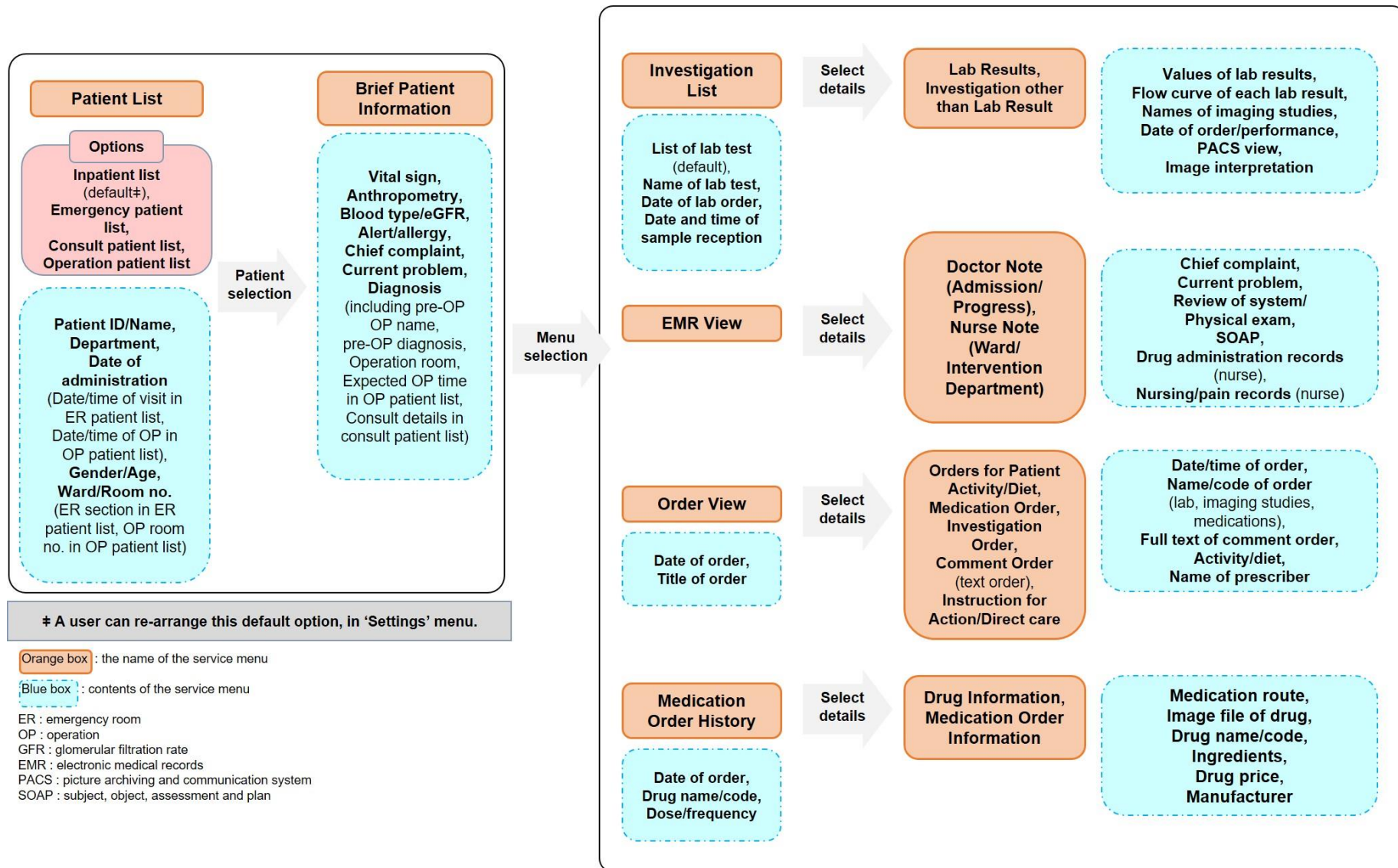

**Service Structure and Contents of the mobile electronic medical record**

Supplement: Multimedia Appendix 1 [file jmir_v19i10e340_app1.pdf]
