# Supplementary material for: What Clinical Information Is Valuable to Doctors Using Mobile Electronic Medical Records and When?
Source: J Med Internet Res. 2017 Oct 18;19(10):e340. doi: 10.2196/jmir.8128 (PMC5666226; doi:10.2196/jmir.8128)
Supplement: Multimedia Appendix 2 [file jmir_v19i10e340_app2.pdf]

Overall usage statistics of the m-EMR based on doctor position.

Variables	General medical departments				Surgical departments				Total		
	Professor (n=131)	Fellows (n=95)	Residents (n=172)	P^a	Professor (n=98)	Fellows (n=66)	Residents (n=91)	P^a	General medical (n=398)	Surgical (n=255)	P^b
**User demographics**											
Mean age in years (SD)	46 (8)	34 (2)	30 (3)	<.001 (*F*)	49 (9)	35 (2)	31 (3)	<.001 (*F*)	36 (9)	39 (10)	.003 (*F*)
Number of male users (%)	47 (12)	97 (24)	76 (19)	<.001 (χ^2)	52 (20)	87 (34)	56 (22)	<.001 (χ^2)	220 (55)	195 (76)	<.001 (χ^2)
Number of female users (%)	48 (12)	34 (9)	96 (24)		14 (5)	11 (4)	35 (14)		178 (45)	60 (24)	
**Usage statistics**											
Total usage count by doctor position	103,411	82,215	209,234		64,283	39,184	26,602		394,860	130,069	
Mean usage count by doctor position (SD)	789 (1678)	865 (1082)	1216 (2131)	.09 (*F*)	656 (1172)	594 (1227)	292 (315)	.03 (*F*)	991 (1787)	510 (986)	<.001 (*F*)
**Mean usage count by menu (SD)**											
Inpatient list	258 (292)	233 (475)	342 (508)	.10 (*F*)	171 (363)	162 (230)	89 (92)	.05 (*F*)	286 (456)	138 (241)	<.001 (F)
Investigation other than lab results	131 (184)	259 (729)	262 (482)	.16 (*F*)	139 (346)	103 (206)	64 (82)	.16 (*F*)	230 (522)	97 (220)	.001 (*F*)
Lab results	119 (179)	133 (260)	176 (248)	.14 (*F*)	94 (206)	85 (147)	46 (51)	.08 (*F*)	149 (238)	73 (142)	<.001 (*F*)
Doctor note	107 (146)	73 (108)	177 (690)	.19 (*F*)	44 (91)	65 (145)	19 (23)	.02 (*F*)	129 (477)	42 (101)	.01 (*F*)
Investigation list	97 (160)	93 (167)	91 (132)	.96 (*F*)	77 (189)	81 (152)	34 (43)	.06 (*F*)	93 (149)	63 (137)	.02 (*F*)

Nurse note	58 (116)	60 (104)	108 (449)	.42 (F)	51 (109)	94 (247)	12 (13)	.02 (F)	82 (318)	53 (165)	.27 (F)
PACS view	52 (85)	69 (196)	46 (70)	.38 (F)	49 (119)	59 (108)	28 (32)	.10 (F)	54 (123)	45 (93)	.36 (F)
Consult patient list	94 (183)	24 (88)	29 (58)	<.001 (F)	23 (117)	24 (44)	2 (2)	.31 (F)	46 (120)	18 (69)	.01 (F)
Order view	29 (38)	24 (39)	40 (81)	.12 (F)	21 (32)	70 (277)	11 (12)	.11 (F)	33 (62)	32 (157)	.95 (F)
Emergency patient list	39 (79)	25 (102)	58 (163)	.16 (F)	12 (19)	10 (16)	13 (15)	.51 (F)	43 (129)	12 (16)	.001 (F)
Operation patient list	3 (5)	3 (4)	6 (11)	.15 (F)	17 (28)	61 (182)	12 (24)	.04 (F)	4 (8)	34 (121)	<.001 (F)
Medication history	3 (4)	4 (10)	4 (6)	.82 (F)	2 (1)	2 (2)	2 (1)	.61 (F)	4 (7)	2 (1)	.07 (F)

[a]Tested null hypotheses: χ^2 test, gender and doctor positions are independent; F test, mean values (age and usage) of doctor's position have the same mean.

[b]Tested null hypotheses: χ^2 test, gender and two departments (general medical vs surgical) are independent; F test, the mean values (age and usage) of the two departments (general medical vs. surgical) are the same.
